# Supplementary material for: Detecting Crystallographic Lattice Chirality using Resonant Inelastic X-ray Scattering
Source: Sci Rep. 2019 Sep 4;9:12771. doi: 10.1038/s41598-019-49157-2 (PMC6726621; doi:10.1038/s41598-019-49157-2)
Supplement: Supplementary file 1 — Supplementary Figures [file 41598_2019_49157_MOESM1_ESM.pdf]

Supplementary Figures - Detecting Crystallographic Lattice Chirality using Resonant Inelastic X-ray Scattering

Sean Mongan[1], Zengye Huang[2], Trinanjan Datta[1,2,*], Takuji Nomura[3], and Dao-Xin Yao[2,*]

[1]Department of Chemistry and Physics, Augusta University, 1120 15th Street, Augusta, Georgia 30912, USA.
[2]State Key Laboratory of Optoelectronic Materials and Technologies, School of Physics, Sun Yat-Sen University, Guangzhou 510275, China.
[3]Synchrotron Radiation Research Center, National Institutes for Quantum and Radiological Science and Technology, SPring-8, 1-1-1 Kouto, Sayo, Hyogo 679-5148, Japan.
[*]tdatta@augusta.edu
[*]yaodaox@mail.sysu.edu.cn

ABSTRACT

The supplementary figures show the scattering geometry, the individual channel contributions arising from transition between the different bands (at zero resolution), and racemic mixture RIXS response including experimental resolution at 10 meV, 15 meV, 50 meV, and 70 meV. The 30 meV resolution results are included in the main article.

Supplementary Figure 1

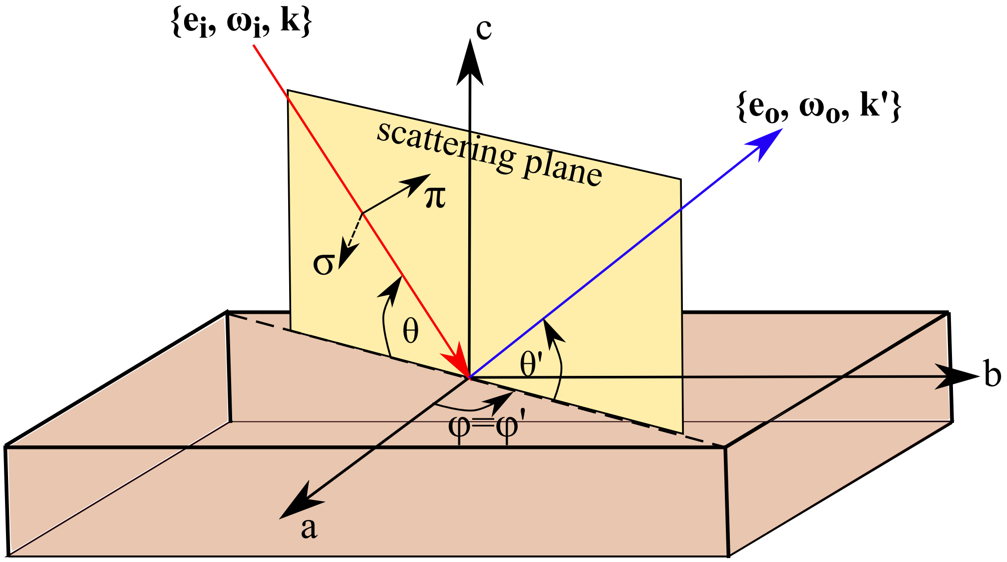

Supplemental Figure S1. Sketch of the scattering geometry displaying the definitions of incoming $\{e_i, \omega_i, \mathbf{k}_i\}$ and outgoing $\{e_o, \omega_o, \mathbf{k}_o\}$ polarization, energy, and wave vector respectively. The scattering plane (yellow) shows the two possible directions of polarization, in-plane polarization (π) and the out-of-plane (σ). The incoming (outgoing) Bragg angles are denoted by $\theta(\theta')$. The in-plane azimuthal angles are given by ϕ and ϕ'.

Supplementary Figure 2

With four participating bands there are sixteen channels. These channels are classified as $m-n$, where $m, n = 1, 2, 3$, and 4. For example, $2-3$ refers to a transition between band number 2 and band number 3.

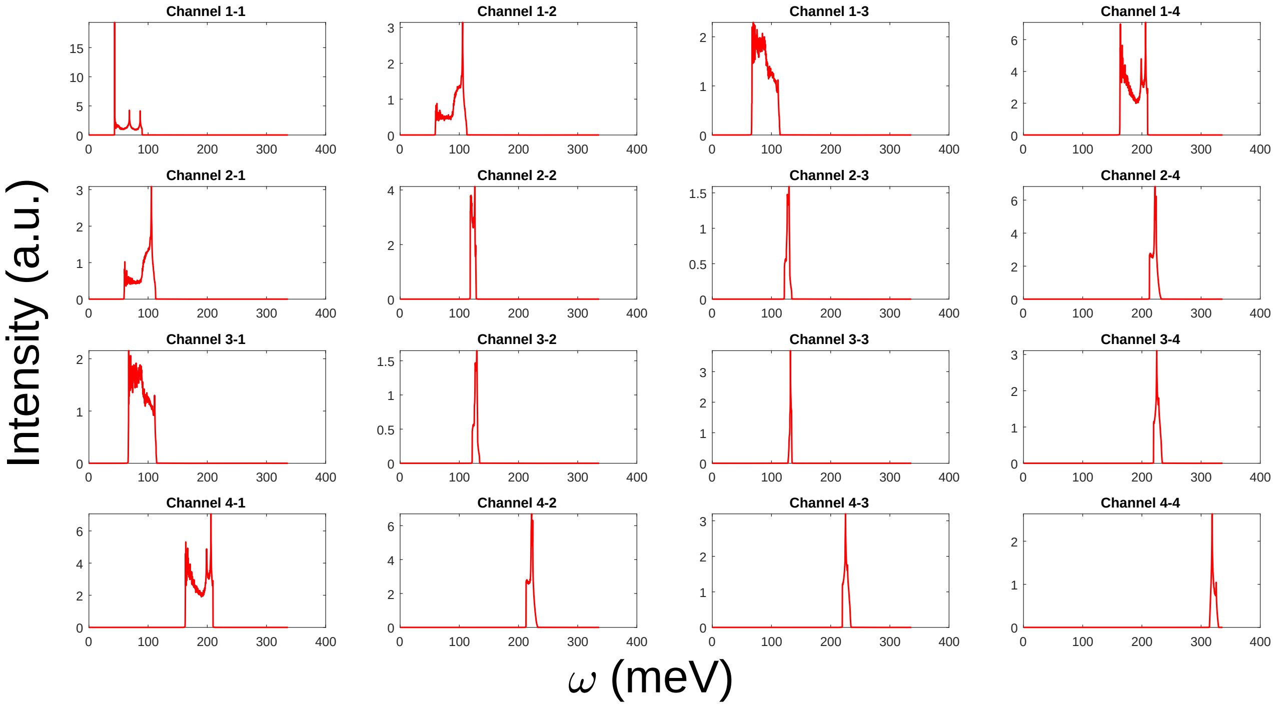

Supplemental Figure S2. RIXS channel responses for the right chiral lattice with the right ordering wave vector. $x-$ axis represents energy in meV. $y-$ axis is normalized intensity.

Supplementary Figure 3

With four participating bands there are sixteen channels. These channels are classified as $m-n$, where $m,n = 1,2,3$, and 4. For example, $2-3$ refers to a transition between band number 2 and band number 3.

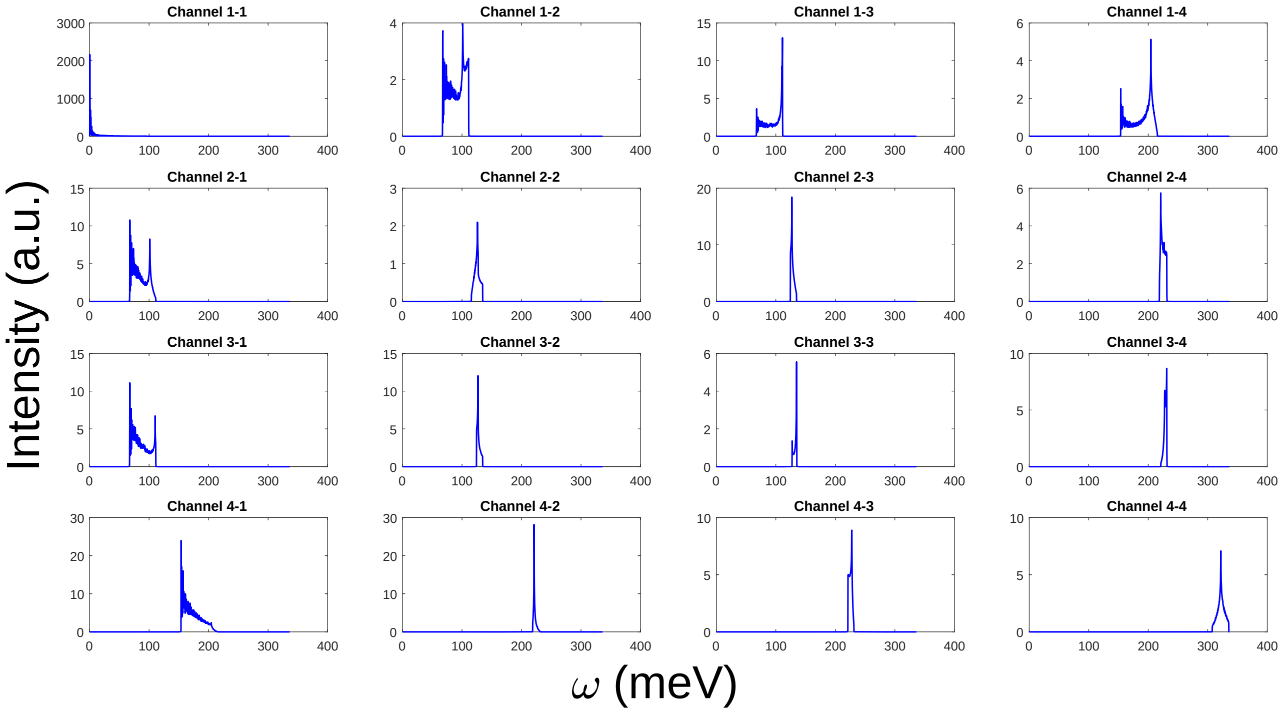

Supplemental Figure S3. RIXS channel responses for the left chiral lattice with the right ordering wave vector. $x-$ axis represents energy in meV. $y-$ axis is normalized intensity. The $i-j$ and $j-i$ channel responses should be symmetric. However, in this case we note a discrepancy which is potentially due to a computational issue, rather than breakdown of any fundamental physical process.

Supplementary Figure 4

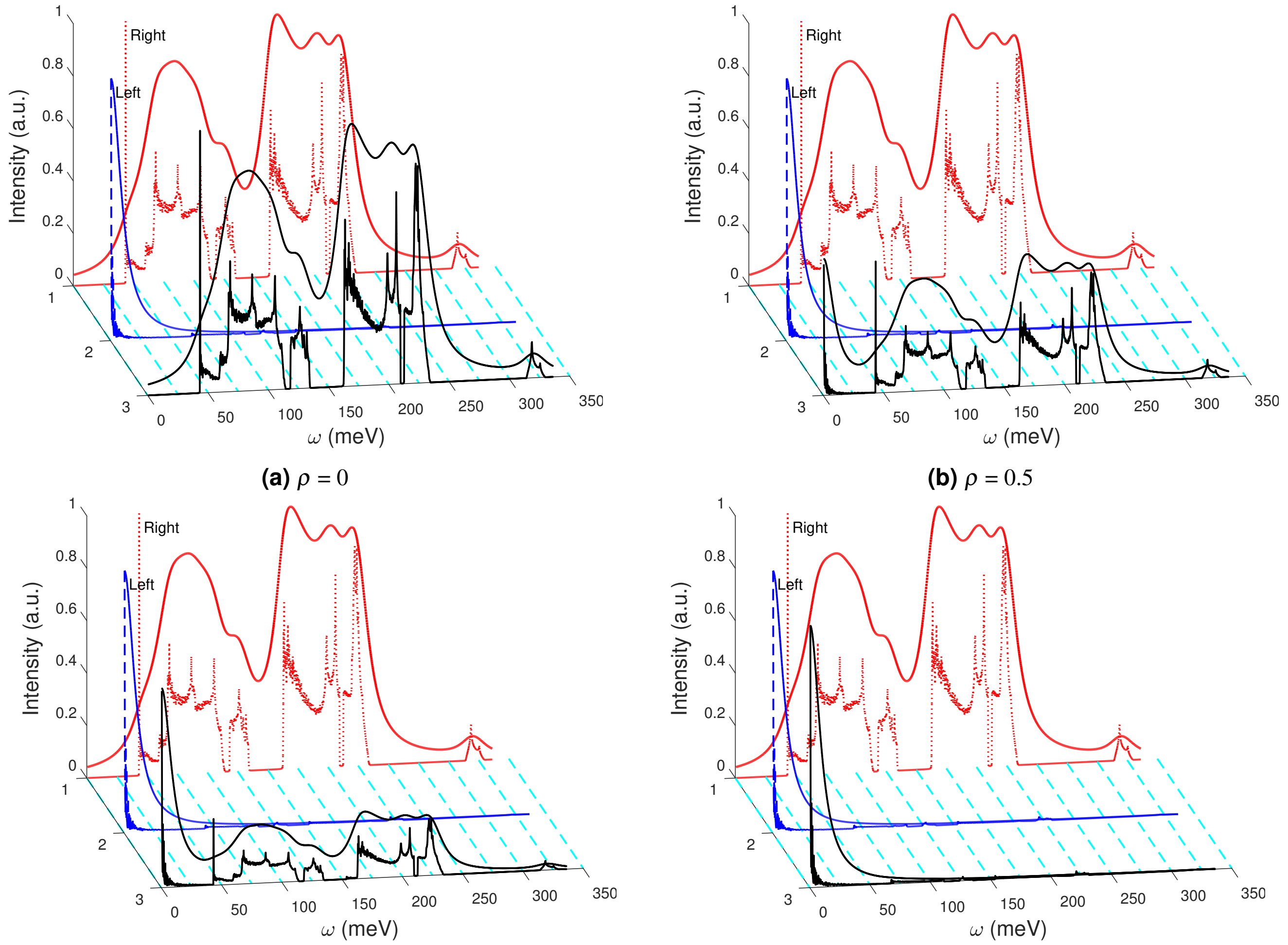

(a) $\rho = 0$ **(b)** $\rho = 0.5$

(c) $\rho = 0.75$ **(d)** $\rho = 1$

Supplemental Figure S4. Chiral composition mixture response for Flack parameter ρ variation with 10 meV resolution. All responses are for the right chiral ordering wave vector and includes the effects of the resonance factor. (a) $\rho = 0.0$ (pure right chiral), (b) $\rho = 0.5$ (racemic conglomerate), (c) $\rho = 0.75$, and (d) $\rho = 1.0$ (pure left chiral).

Supplementary Figure 5

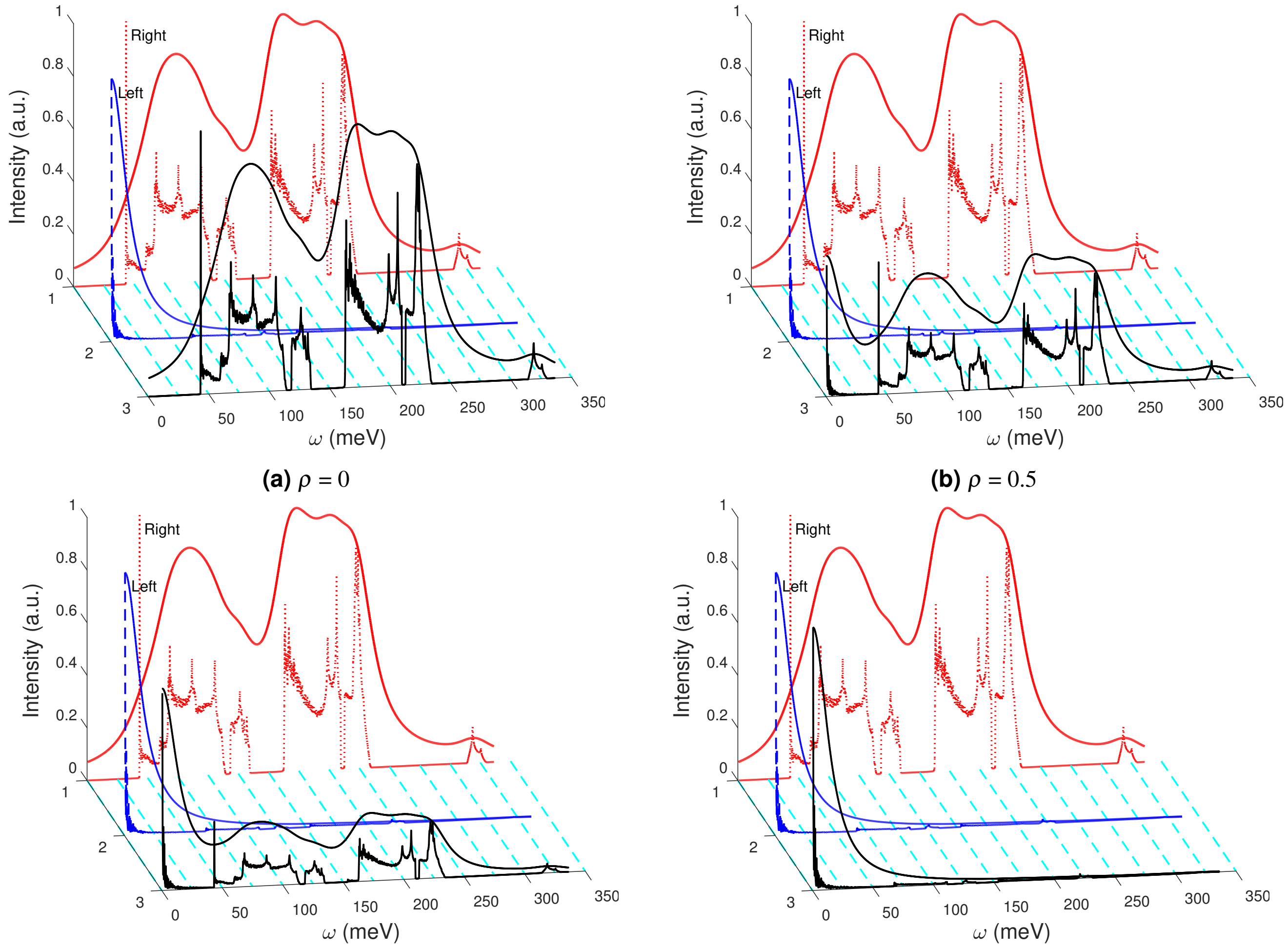

(a) $\rho = 0$ **(b)** $\rho = 0.5$

(c) $\rho = 0.75$ **(d)** $\rho = 1$

Supplemental Figure S5. Chiral composition mixture response for Flack parameter ρ variation with 15 meV resolution. All responses are for the right chiral ordering wave vector and includes the effects of the resonance factor. (a) $\rho = 0.0$ (pure right chiral), (b) $\rho = 0.5$ (racemic conglomerate), (c) $\rho = 0.75$, and (d) $\rho = 1.0$ (pure left chiral).

Supplementary Figure 6

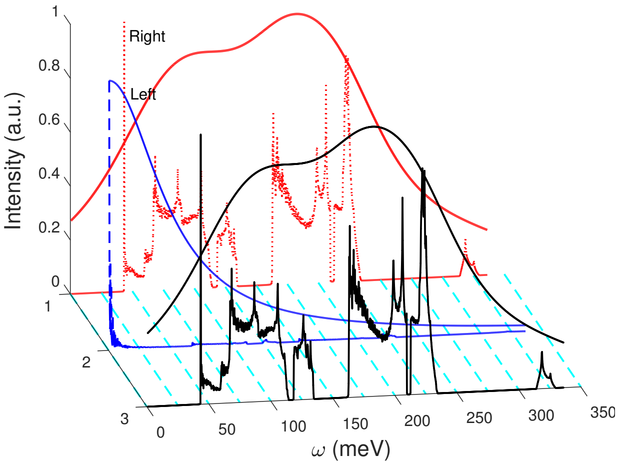

(a) $\rho = 0$

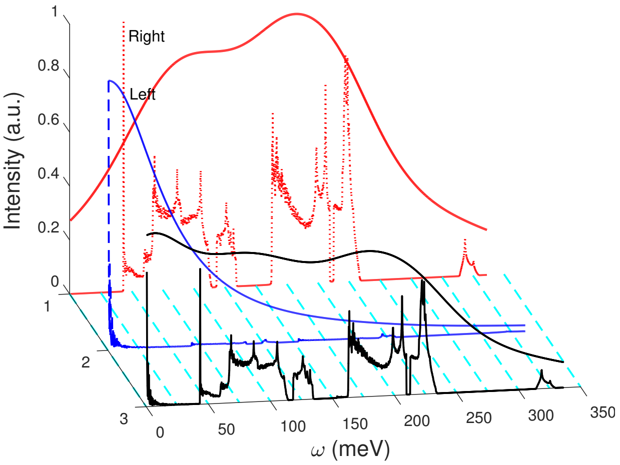

(b) $\rho = 0.5$

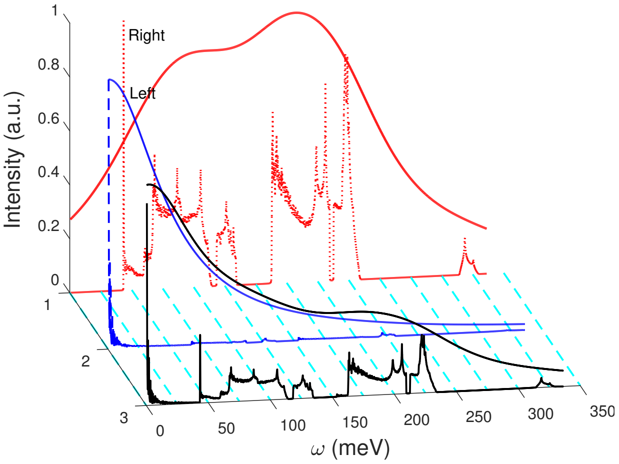

(c) $\rho = 0.75$

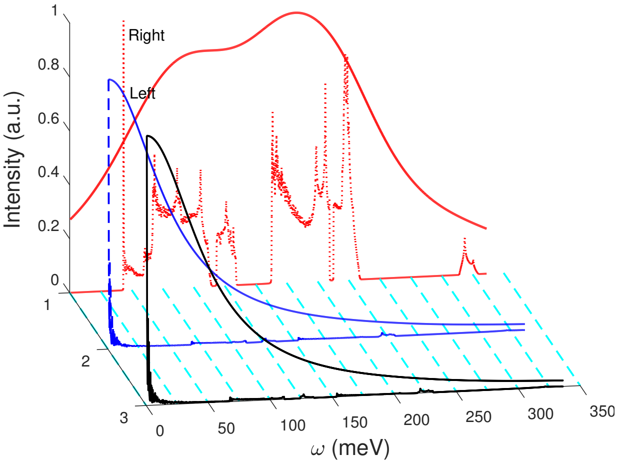

(d) $\rho = 1$

Supplemental Figure S6. Chiral composition mixture response for Flack parameter ρ variation with 50 meV resolution. All responses are for the right chiral ordering wave vector and includes the effects of the resonance factor. (a) $\rho = 0.0$ (pure right chiral), (b) $\rho = 0.5$ (racemic conglomerate), (c) $\rho = 0.75$, and (d) $\rho = 1.0$ (pure left chiral).

Supplementary Figure 7

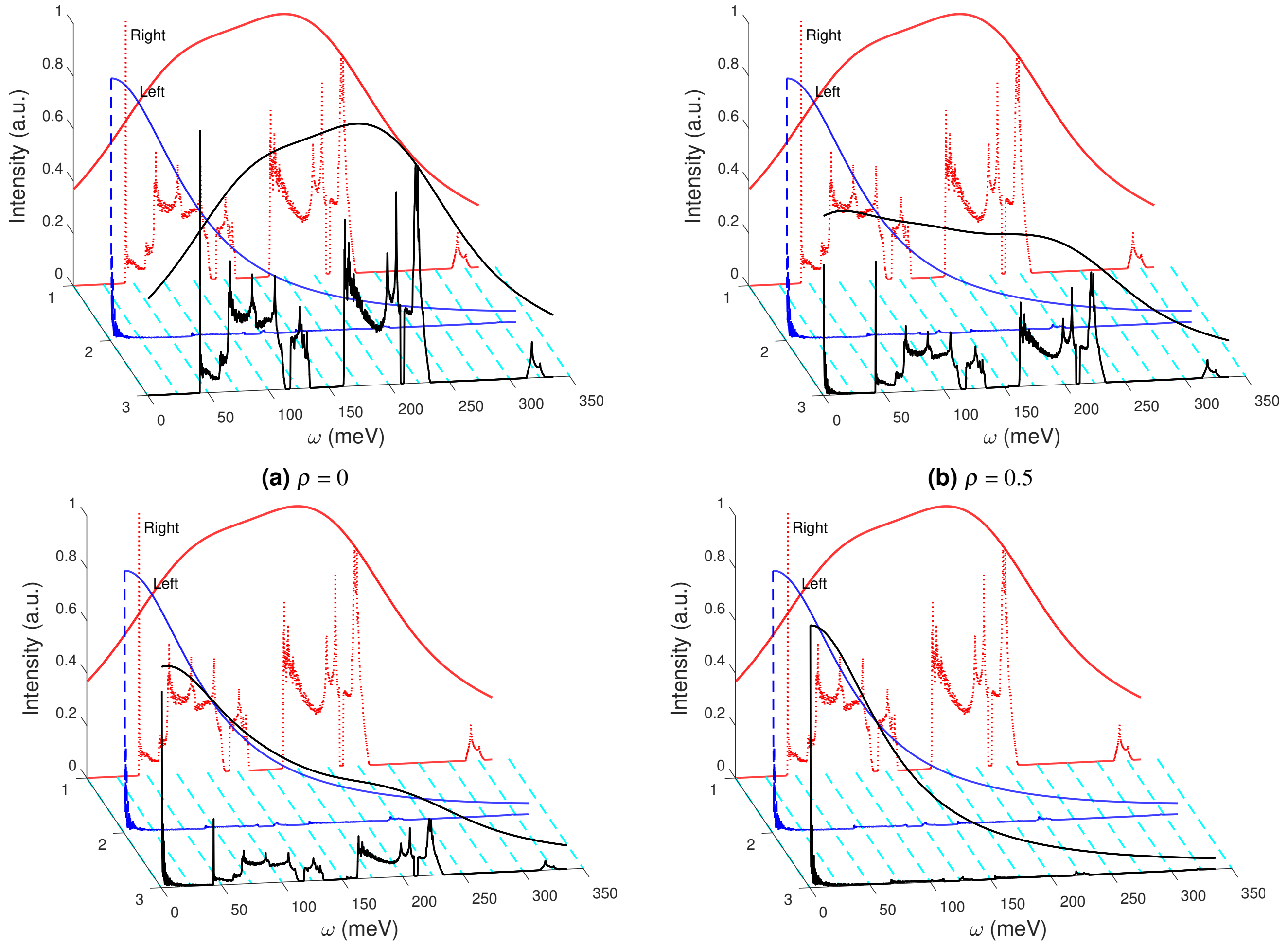

(a) $\rho = 0$ **(b)** $\rho = 0.5$

(c) $\rho = 0.75$ **(d)** $\rho = 1$

Supplemental Figure S7. Chiral composition mixture response for Flack parameter ρ variation with 70 meV resolution. All responses are for the right chiral ordering wave vector and includes the effects of the resonance factor. (a) $\rho = 0.0$ (pure right chiral), (b) $\rho = 0.5$ (racemic conglomerate), (c) $\rho = 0.75$, and (d) $\rho = 1.0$ (pure left chiral).
